# Biofilm forming properties of quinolone resistant *Escherichia coli* from the broiler production chain and their dynamics in mixed biofilms

**DOI:** 10.1186/s12866-020-01730-w

**Published:** 2020-03-04

**Authors:** Live L. Nesse, Ane Mohr Osland, Solveig S. Mo, Camilla Sekse, Jannice S. Slettemeås, Anna Eline E. Bruvoll, Anne Margrete Urdahl, Lene K. Vestby

**Affiliations:** Norwegian Veterinary Institute, P.O. Box 750 Sentrum, N-0106 Oslo, Norway

**Keywords:** Biofilm, Quinolone resistance, *E. coli*, QREC, Broiler chicken

## Abstract

**Background:**

Quinolone resistant *Escherichia coli* (QREC) have been found in samples from Norwegian broiler chicken, despite quinolones not being administered to poultry in Norway. Biofilm production may be one factor contributing to the observed persistence in the broiler production chain. In the present study, 158 QREC strains from chicken caecal and retail meat samples were screened for biofilm production in microtiter plates, biofilm morphotype on Congo Red (CR) agar plates and phylotype by multiplex PCR. Furthermore, the dynamics in mixed biofilms with strains of different morphotypes were studied on glass slides and on CR agar plates.

**Results:**

All strains but one produced biofilm in microtiter plates and/or on CR agar plates at room temperature. There were no differences between strains from chicken caecum and chicken retail meat in the mean amount of biofilm produced in microtiter plates. Furthermore, no differences in biofilm production were observed between phylotypes. However, significant differences in biofilm production were found between biofilm morphotypes. The morphotype RDAR (red dry and rough, which has both curli and cellulose in the matrix, was displayed by 70% of the strains. Mean biofilm production by these strains were significantly higher than by strains with the morphotypes PDAR (pink dry and rough) with only cellulose or BDAR (brown dry and rough) with only curli. Interestingly, the two latter morphotypes produced biofilms with the morphotype RDAR when grown together. None of the strains achieved significantly higher numbers of colony forming units (cfu) in mixed biofilms than in single strain biofilms on glass slides.

**Conclusions:**

The results indicate that QREC can form biofilm reservoirs on both inert and organic surfaces in production environments, as well as on meat. This may contribute to persistence and dissemination of the strains. Strains with both curli and cellulose in the biofilm matrix were significantly better biofilm formers than strains lacking one of these components. However, strains with only one of the components could compensate for this by producing mixed biofilms with strains having the other component, and thereby most likely enhance their probabilities of persistence in the production environment.

## Background

Quinolones is a group of antimicrobials considered to be critically important for human medicine and should preferably be reserved for treatment of severe infections in humans [1]. In Norwegian livestock production, the use of quinolones has been very low, i.e. only 10–15 kg of active substance of fluoroquinolones per year [2], and quinolone resistant bacteria from production animals has usually been a rare finding. However, after the implementation of a selective method in the Norwegian monitoring program for antimicrobial resistance in animals, food and feed (NORM-VET), it was shown that quinolone resistant *Escherichia coli* (QREC) were present at low levels in a high proportion of the samples from broiler chicken [3, 4]. As quinolones are not administered to poultry in Norway, little is known on how, why, when and where this resistance has developed, and which factors that may contribute to persistence and dissemination of QREC in the broiler production chain.

Production of biofilm may be one such factor. Biofilms are defined as bacterial populations adherent to each other and/or surfaces or interfaces, and enclosed in a self-produced matrix [5, 6]. The composition of the matrix differs depending on the species involved in biofilm formation and on the environment, but it often consists of proteins, polysaccharides, and/or extracellular DNA. Biofilms can accumulate on a wide variety of substrates. Bacteria in biofilms are more tolerant to disinfectants, antimicrobial agents and most other forms of environmental stress, compared to their planktonic counterparts [7–11]. Not surprisingly, biofilms are renowned for the problems they cause in clinical settings, food production facilities, and industrial plants [12, 13].

Previous studies have shown variations in the ability of different *E. coli* strains to produce biofilm under conditions relevant for the food production chain [14, 15]. *E. coli* strains also display variations in biofilm matrix composition which can be visualized when grown on agar plates with Congo Red and Coomassie Blue dyes (CR agar plates) [16, 17]. Three different biofilm morphotypes can be observed; RDAR (red, dry and rough) expressing both cellulose and curli fimbriae (curli), PDAR (pink, dry and rough) expressing cellulose and BDAR (brown, dry and rough) expressing curli. A fourth morphotype SAW (smooth and white) indicates no biofilm growth. The RDAR morphotype is the most common and the best studied [16]. Little is known on whether morphotype affects the amount of biofilm produced.

The aim of the present study was to characterize the biofilm forming abilities of QREC from Norwegian broiler production to elucidate a possible role of biofilm formation in persistence in production environments, as well as in contamination of meat. Furthermore, we aimed to study the dynamics in mixed biofilms with strains of different biofilm morphotypes to see whether these strains would gain benefits by producing such biofilms.

## Results

### Screening of biofilm production, morphotypes and phylotypes

Large variations in biofilm formation in microtiter plates were observed between strains, with A_595_ values ranging from − 0.027 to 3.333, and a mean A_595_ of 1.215 (Table 1). In total, 84.2% of the strains were considered positive for biofilm production, i.e. displayed A_595_ values above three standard deviations of the negative controls (A_595_ cut-off = 0.085) (Table S1).

**Table 1:** Distribution of morphotypes in the CR agar plate assay, and biofilm production by the different morphotypes as indicated by A_595_ in the microtiter plate assay. Means with same letter are not statistically different (*p* > 0.05)

Morpho-type	No	% of all	Mean A_595_ ± SD	Min. A_595_	Max. A_595_
RDAR	110	69.6	1.505^A^ ± 0.964	−0.002	3.333
BDAR	42	26.6	0.610^B^ ± 0.764	−0.027	2.744
PDAR	5	3.2	0.143^B^ ± 0.120	0.010	0.261
SAW	1	0.6	0.083	–	–
ALL	158	100.0	1.215 ± 1.000	−0.027	3.333

*Min*. Minimum, *Max*. Maximum, *SD* Standard deviation.

All strains but one produced biofilm on CR agar plates. The dominating biofilm morphotype was RDAR, which was displayed by 69.6% of the strains (Table 1). The morphotypes BDAR and PDAR were displayed by 26.6 and 3.2%, respectively, whereas one strain (0.6%) displayed the non-biofilm morphotype SAW. Mean biofilm production in microtiter plates was significantly higher in RDAR strains than in BDAR (*p* = 1.2*10^− 7^) and PDAR strains (*p* = 0.0018) (Table 1). RDAR strains displayed both a higher percentage of positive biofilm producers, and a higher mean A_595_ of the biofilm positive strains (Table S1).

Phylotype B2 was the most common (47%), followed by D (24%), B1 (16%) and A (13%). There were no significant differences in mean biofilm production in microtiter plates between phylotypes in the total material (Table 2). However, significant differences between phylotypes were observed within morphotypes RDAR and BDAR. Furthermore, biofilm production was displayed by all BDAR strains with phylotypes A and B1, but only by 63.6 and 18.8% of BDAR strains with the phylotypes B2 and D, respectively. Distributions of morphotypes varied between phylotypes (Figure S1). RDAR dominated within phylotypes A and B2 (76.2 and 77.9% of the strains, respectively), whereas a more equal distribution of RDAR and BDAR strains were observed within phylotypes B1 (56.0% vs 44.0%) and D (57.9% vs 42.1%).

**Table 2:** Distribution of phylotypes, and mean biofilm production by the different phylotypes as indicated by A_595_ in the microtiter plate assay, in total material and within morphotypes RDAR and BDAR. Means with same letter are not statistically different (*p* > 0.05)

	Total			RDAR			BDAR		
Phylo-type	No	%	Mean A_595_ ± SD	No	%	Mean A_595_ ± SD	No	%	Mean A_595_ ± SD
A	21	13.3	1.345 ± 1.114	16	14.5	1.503^AC^ ± 1.158	4	9.5	1.029^A^ ± 0.877
B1	25	15.8	1.157 ± 0.821	14	12.7	1.245^A^ ± 0.896	11	26.2	1.046^A^ ± 0.741
B2	74	46.8	1.129 ± 0.862	58	52.7	1.341^A^ ± 0.823	11	26.2	0.458^D^ ± 0.559
D	38	24.1	1.350 ± 1.274	22	20.0	2.105^BC^ ± 1.017	16	38.1	0.311^D^ ± 0.753

*SD* Standard deviation

When comparing strains originating from chicken caecum and retail chicken meat, there were no significant differences in mean biofilm formation (Table 3) or the percentage of biofilm producers in the microtiter plate assay (89,4 and 83.6%, respectively, chi square test *p* = 0.31). Neither were there any differences in distribution of biofilm morphotypes or phylotypes (Table 3 and S2). However, within the most common morphotype RDAR, caecal strains displayed a higher mean biofilm A_595_ than those from meat (Table 3). Also within phylotype D, mean biofilm production was significantly higher in caecal samples than in meat samples (Table S2).

**Table 3:** Comparison of biofilm production by isolates from chicken caecal and retail meat samples as indicated by A_595_ in the microtiter plate assay, in the total material and within morphotypes

	Chicken caecal samples			Chicken retail meat samples			
Morpho-type	No	%	Mean A_595_ ± SD	No	%	Mean A_595_ ± SD	*p* -value*
RDAR	57	67.1	1.677 ± 1.002	53	72.6	1.320 ± 0.895	0.04
BDAR	25	29.4	0.624 ± 0.757	17	23.3	0.591 ± 0.797	0.25
PDAR	3	3.5	0.100 ± 0.140	2	2.7	0.209 ± 0.064	0.80
SAW	0	0.0		1	1.4	0.083	–
ALL	85	100.0	1.312 ± 1.057	73	100.0	1.103 ± 0.924	0.16

*SD* Standard deviation.

* Mean A_595_ of caecal samples vs retail meat samples

### Single and mixed biofilms on glass slides

Six strains, two of each of the morphotypes RDAR, BDAR and PDAR, were used in these experiments (Table 4). These strains displayed relatively large differences in the total number of cfu in the biofilms when grown as single biofilms on glass slides. Furthermore, all except RDAR-1 had similar numbers of cfu in planktonic phase in the growth medium after incubation (Table 4). The number of cfu in the biofilms was not correlated to the number of planktonic cfu.

**Table 4:** Mean log_10_ of total cfu with standard deviation for each strain in the biofilm and in the planktonic fraction in the glass slide assay

Strain no.	Source	Phylo-type	Strain name	Morpho-type	Biofilm log_10_ cfu	Planktonic log_10_ cfu
2014-01-2363	Caecal	A	RDAR-1	RDAR	5.82 ± 0.04	8.65 ± 1.34
2014-01-6040	Caecal	D	RDAR-2	RDAR	6.07 ± 0.16	9.59 ± 0.02
2014-01-5914-1	Meat	B2	PDAR-1	PDAR	6.31 ± 0.15	9.17 ± 0.34
2014-01-7342-1	Meat	B2	PDAR-2	PDAR	6.12 ± 0.0	9.34 ± 0.03
2014-01-7046-1	Caecal	A	BDAR-1	BDAR	4.44 ± 0.06	9.46 ± 0.12
2014-01-2069-1	Caecal	B1	BDAR-2	BDAR	5.07 ± 0.66	9.47 ± 0.12

Twelve different pairs were made by combinations of these strains to be used in studies on dual-species biofilms (Table S3). Each pair contained strains with different morphotypes. Both within the biofilm and in the planktonic phase, the mean total log_10_ cfu after incubation was higher when testing pairs of strains than when testing single strains, despite the total inoculum being the same in both (Table S4). The difference was statistically significant in the planktonic phase (*p* = 0.01), but not in the biofilm (*p* = 0.17). None of the strains displayed higher numbers of cfu within mixed than in the single biofilms. In fact, the mean log_10_ cfu for the PDAR strains, as well as for the RDAR-1 strain, were significantly lower in mixed biofilms with other morphotypes than in single strain biofilms (Table S5).

**Fig. 1:**
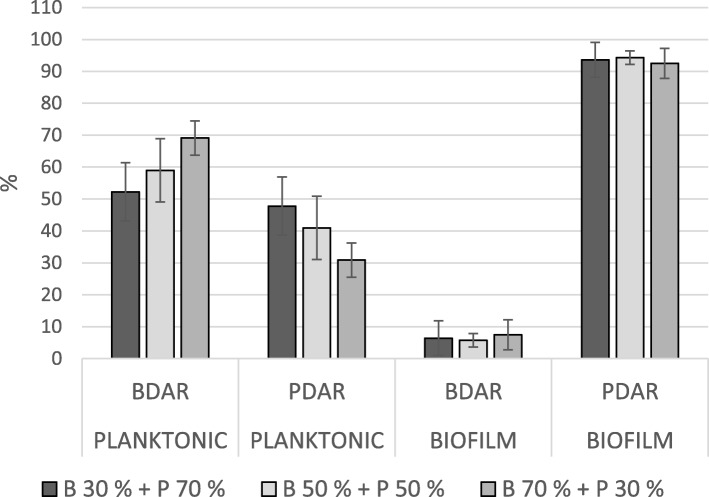
Mean ratio of BDAR and PDAR strains in the planktonic fraction and in the biofilm after incubation, in the glass slide assay. Bars indicate standard deviation. B = BDAR, P = PDAR, the percentages show the inoculation ratio

**Fig. 2:**
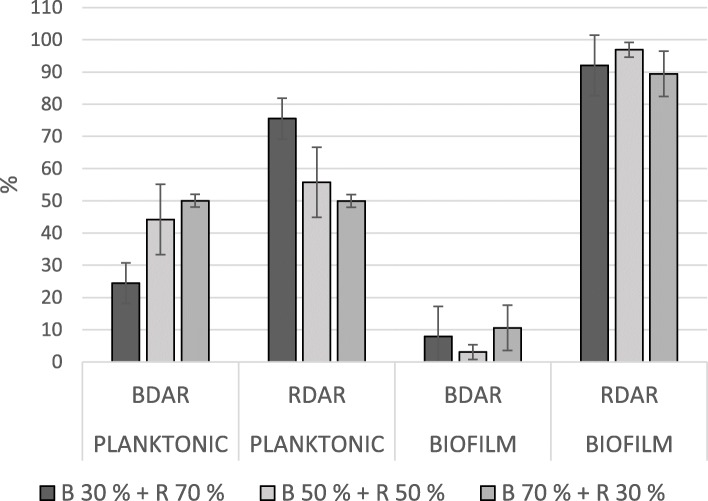
Mean ratio of BDAR and RDAR strains in the planktonic fraction and in the biofilm after incubation, in the glass slide assay. Bars indicate standard deviation. B = BDAR, R = RDAR, the percentages show the inoculation ratio

**Fig. 3:**
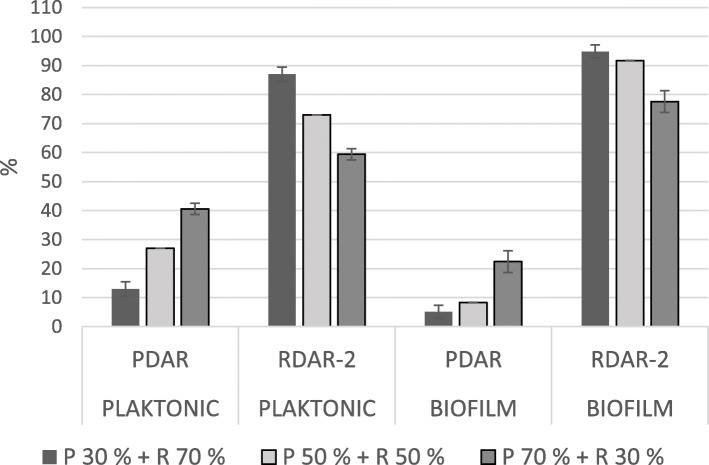
Mean ratio of PDAR strains and RDAR-2 strain in the planktonic fraction and in the biofilm after incubation, in the glass slide assay. Bars indicate standard deviation. P = PDAR, R = RDAR, the percentages show the inoculation ratio

**Fig. 4:**
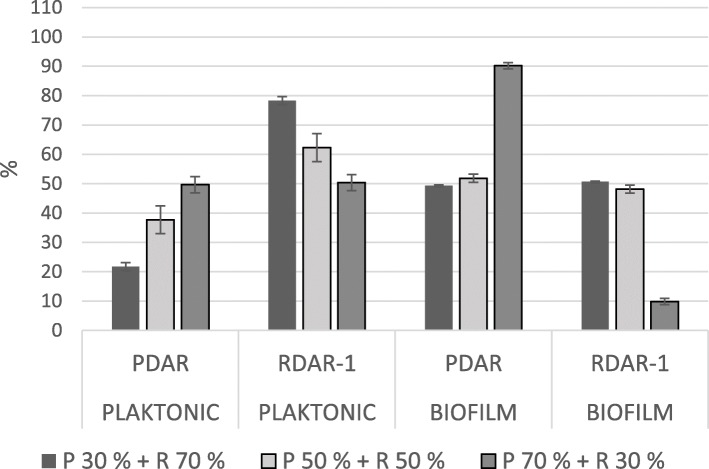
Mean ratio of PDAR strains and the RDAR-1 strain in the planktonic fraction and in the biofilm after incubation, in the glass slide assay. Bars indicate standard deviation. P = PDAR, R = RDAR, the percentages show the inoculation ratio

In mixed biofilms, BDAR strains constituted less than 10% of the total cfu in all pairs, regardless of the ratio of the inoculum (Figs. 1 and 2). In these pairs, the ratio in the biofilm corresponded with the strains’ relative biofilm production as single strains. The ratios observed in pairs with one RDAR and one PDAR strain depended on which RDAR strain was included. The strain RDAR-2 dominated in both pairs with PDAR-strains (Fig. 3), although these three strains all produced biofilms of the same magnitude when inoculated alone. In the biofilms where PDAR-strains were paired with the strain RDAR-1, the ratio seemed to reflect a combination of inoculation ratio and relative biofilm forming abilities as single strains (Fig. 4).

In the planktonic phase, the cfu ratio of each pair of strains after incubation reflected the ratio inoculated (Fig. 1, 2, 3 and 4). In addition, the BDAR strains outcompeted the PDAR strains, and the two RDAR strains outcompeted both the PDAR and the BDAR strains even though the strain RDAR-1 had the lowest growth rate when inoculated alone.

### Single and mixed biofilms on CR agar plates

**Fig. 5:**
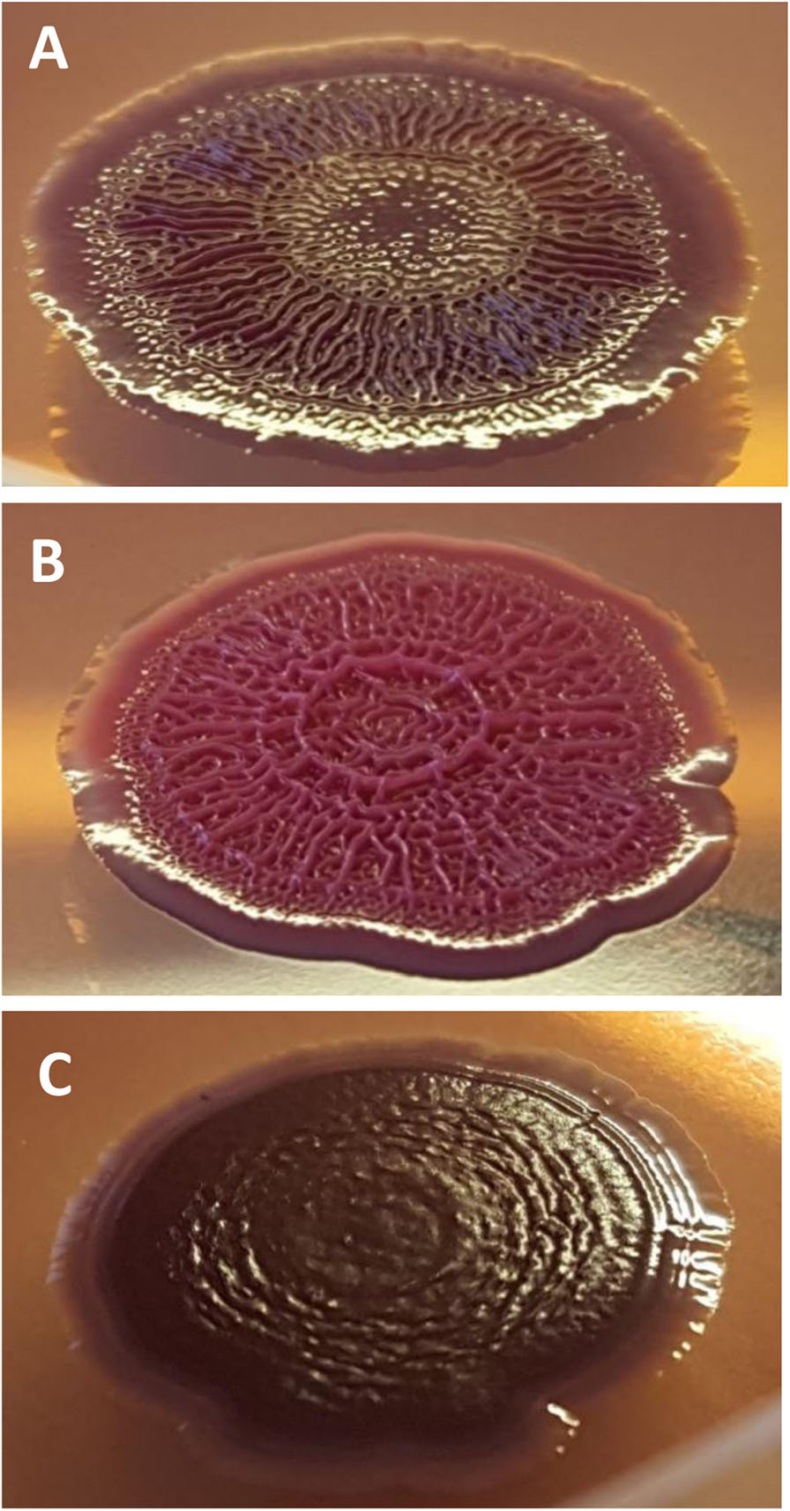
Morphology on CR agar plates. **a** RDAR-2 (red, dry and rough). **b** PDAR-1 (pink, dry and rough). **c** BDAR-2 (brown, dry and rough)

All six strains tested displayed typical morphotypes when grown as single biofilms on CR agar plates (Fig. 5). The RDAR and PDAR strains, which both produced cellulose as part of the matrix, displayed highly structured biofilms with wrinkles and ridges. RDAR and BDAR strains, which both had curli fimbriae in the matrix, produced dark red/brown biofilms.

**Fig. 6:**
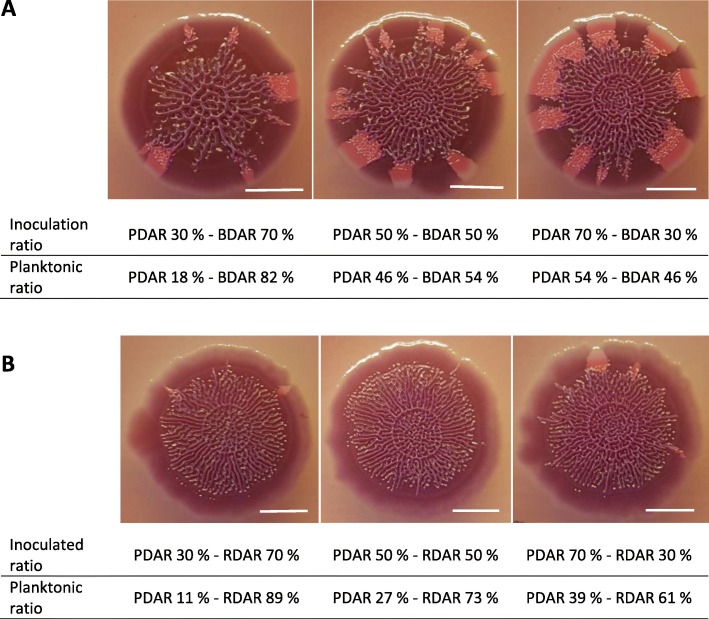
Biofilms on CR agar plates produced by inoculation of pairs of strains in different ratios, and incubating at 20 °C for 7 days. Planktonic ratio was obtained in the glass slide assay. **a** PDAR-1 (pink) and BDAR-2 (brown). **b** PDAR-1 and RDAR-2 (dark red). Bar indicates 4 mm

All inoculated pairs formed biofilms with a combination of structure and color indicating the presence of both cellulose and curli fimbriae during the first 3 days. After this time, the strains started expanding in a spatial fashion displaying their original morphotypes. For all pairs except those including RDAR-2, the ratio of the strains during spatial growth reflected the ratio that was displayed in the planktonic phase in the glass slide assay (Fig. 6a). In contrast, the RDAR-2 strain dominated in all pairs, just as it did in biofilm on glass slides (Fig. 6b).

## Discussion

In the present study, we show that the majority of QREC from the Norwegian broiler production chain produced biofilm at room temperature in the microtiter plate assay, and that all but one strain produced biofilm in the CR agar plate assay. The results show that QREC can form biofilm both on inert and organic surfaces. Strong biofilm producing abilities have earlier been shown to correlate with long-term persistence in various production environments [12, 17]. Consequently, our results indicate that QREC biofilms may be formed and act as reservoirs contributing to the observed persistence and dissemination of QREC in the broiler production chain. In the total material, there was no significant difference between caecal and meat strains in biofilm production in the microtiter plate assay. Although the microtiter plate assay might not reflect the true biofilm forming abilities of the strains on organic surfaces like chicken meat, the results nevertheless show that these strains have the capacity to be good biofilm formers. Furthermore, the fact that more strains produced biofilm on the organic surface of CR agar plates than in microtiter plates under the same conditions, indicate that these strains may colonize meat by producing biofilms.

Biofilm formation in microtiter plates was strongly influenced by the presence of cellulose and curli in the matrix, as indicated by the morphotype on CR agar plates. The most common morphotype RDAR, which had both components, produced significantly more biofilm than PDAR and BDAR having only one of these components. The RDAR strains displayed both a higher percentage of biofilm formers in microtiter plates and a higher mean biofilm production by these strains. Both curli and cellulose are known to determine the complex macroscopic architecture of the biofilm [18]. Curli are aggregative amyloid fibers that are involved in adhesion to surfaces and cell aggregation [19], including adherence to avian intestinal cells, and persistence in the caecum of chickens [20]. However, both a synergistic [19] and a counteractive [21] role of cellulose in curli-mediated cell adherence and colonization of solid surfaces has been suggested. Our results strongly indicate that the presence of curli and cellulose together contributes to increased biofilm production, probably by promoting adhesion, as well as biofilm build up once adhered. Interestingly, similar variations between strains in the amount of biofilm produced were not observed when the they were allowed to form biofilm on CR agar plates. One explanation may be that the ability to adhere to the surface was less critical in this test system. It may also be that other structures are more important for adhesion to organic surfaces like the CR agar. Both other fimbria and exopolymers, as well as flagella, autotranspoters and other proteinaceous adhesions expressed by *E. coli* have been shown to contribute to adhesion to organic material such as mammal cells and plants (reviewed by [22]).

All our PDAR strains were poor biofilm producers in the microtiter plate assay, although production of cellulose has been reported to enhance bacterial adherence [23]. Interestingly, the two strains chosen for further testing turned out to be excellent biofilm producers on glass slides. This was unexpected because our previous work suggested a good correlation between *E. coli* biofilm formation in microtiter plates and on glass slides [14]. However, in that study the morphotypes were unknown, and as PDAR strains seems to be rare, there might not have been any among the strains tested. Consequently, the observed variation in the ability to form biofilm on different surfaces may be a special feature for the PDAR morphotype. This implies that cellulose does play a role in the adhesion to some surfaces, but not to all.

No difference in mean biofilm production was observed between phylotypes A, B1, B2 and D when all strains were included. However, significant differences between phylotypes were observed within morphotypes RDAR and BDAR. These results indicate a genetic association to a lower level of characterization than phylotype. This level may possibly be serogroup or serotype, as associations between biofilm forming abilities and serotypes of *E. coli*, as well as serovar of *Salmonella enterica,* have been described previously [14, 17].

To see whether strains with different morphotypes would benefit from producing biofilm together, we studied the dynamics of different combinations in mixed biofilms. In particular, we wanted to see whether PDAR and BDAR strains would complement each other by producing a common matrix with both cellulose and curli, i.e. the RDAR morphotype, which is believed to play an important role in the survival of the bacteria in the environment [24]. Indeed, all the pairs produced biofilms displaying the RDAR morphotype during the first 3 days on CR-agar. This showed that PDAR and BDAR strains are able to cooperate on making a biofilm with a matrix that probably enhances their chances of persistence.

After 3 days, further expansion of all the mixed biofilms on CR agar plates occurred with the strains growing in a spatial manner displaying their original morphotypes. It has been suggested that onset of exploitative competition can be expected when mixed strains with high metabolic overlap reach a high density relative to available sources [25]. When a biofilm on agar reaches this stage, such competition is most likely to occur at the edge of the biofilm, which is the only place where the bacteria can gain sustained access to nutrients for cell division [26, 27]. In our experiments, the observed ratios of the pairs at the biofilm edges were in most cases similar to the ratios displayed when the same pairs when grown together in planktonic solution. This observation supports the notion that cell growth and division is an important competitive factor at this stage of biofilm production, thereby contributing to the spatial organization of the strains. One strain, i.e. the RDAR-2 strain, behaved differently by dominating the growing edge in all pairs. The reason is most likely that it had already outcompeted the other strains during the first 3 days, just as it did in all pairs in the glass slide assay. When biofilms are grown on CR agar pates, the surface is the only access to nutrition. On other surfaces and in other environments, different parts of the biofilm may have a more direct access to nutrition, and this may alter the dynamics.

None of the strains achieved significantly higher numbers of cfu in the mixed biofilms on glass slides as compared to single strain biofilms. Thus, there was no observed quantitative benefits for the strains of being in a mixed biofilm. This is in contrast to earlier observations of *E. coli* in mixed biofilms with *Acinetobacter calcoaceticus,* where the biovolume of *E. coli* O157:H7 was 400-fold higher in a dynamic co-cultured biofilm than in a monoculture [28]. Also, dual-species biofilms have been found to favor *Salmonella* compared to *Salmonella* in mono-species biofilms, with biovolume increases of 2.8-fold and 3.2-fold in the presence of *Staphylococcus* and *Pseudomonas*, respectively [29]. It may be that the strains in our study, being of the same species, were too similar to benefit from such mutualism or synergy [25].

For most pairs, the ratios of the strains in two-day-old biofilms on glass slides reflected the relative biofilm forming abilities of the strains. This was in contrast to the planktonic fraction where all the strains, to some extent, competed with each other. These results are in agreement with an earlier study where *Salmonella* ser. Typhimurium strains outcompeted *E. coli* strains in the planktonic growth phase, whereas development in mixed biofilm was highly dependent upon the strains’ biofilm properties [30]. Similar results have also been obtained in a study with uropathogenic and non-pathogenic *E. coli* in mixed biofilms [31].

## Conclusions

The majority of QREC isolated from Norwegian broiler production formed biofilm at room temperature, both on an inert and an organic surface. These results indicate that QREC can form biofilm reservoirs in production environments that may contribute to persistence and dissemination of these strains. Biofilm forming abilities did not differ between caecum and meat samples, indicating that biofilm may also facilitate persistence on meat. This is supported by the observation that all but one strain produced biofilm on an organic surface like CR-agar. Strains displaying both curli and cellulose in the biofilm matrix were the best biofilm formers. Strains lacking one matrix component could compensate for this by producing mixed biofilms with strains having that component, thereby most likely enhancing their probabilities of persistence in the production environment.

## Methods

### Bacterial isolates

The QREC strains were originally collected as part of the NORM-VET program in 2014 [4], and originated from broiler caecal samples (*n* = 85) and retail chicken meat (*n* = 73).

All strains were stored at − 80 °C in brain heart infusion broth (BHI; Difco, BD, Franklin Lakes, NJ, USA) supplemented with 15% glycerol (Merck KGaA, Darmstadt, Germany) and recovered on blood agar (sheep blood) at 37.0 ± 1.0 °C overnight. The bacterial cultures were then transferred into Luria-Bertani broth (LB; Merck) and incubated statically overnight at 37.0 ± 1.0 °C. LB without NaCl (LB^wo^/NaCl; Bacto-tryptone 10 g/liter, yeast extract 5 g/liter) was used as the test broth in the biofilm assays.

### Determination of phylotypes

All isolates were subjected to phylotyping using multiplex PCR as described previously [32]. The isolates were classified into phylotype A, B1, B2 or D. An isolate belonging to the B2 group, (*E. coli*2003500827) [33] producing amplicons with all four primer sets, was included as positive control in each PCR run and milli-Q water was used as negative control.

### Biofilm production on polystyrene (microtiter plate assay)

Biofilm production on polystyrene was measured in the microtiter plate assay and performed as described previously [14] using 96-well Nunc™ Nunclon™ microtiter plates (Nunc A/S, Roskilde, Denmark). In short, 30 μL of an overnight culture were added to 100 μL LB^wo^/NaCl in three parallel wells of a microtiter plate. The microtiter plates were incubated statically for 2 days at 20.0 ± 1.0 °C. Room temperatures are generally 10–16 °C during slaughter and 8–10 °C during further processing. In chicken houses, the temperature is gradually decreased to 20 °C as the chicken grow. Based on earlier studies, the chosen test temperature and incubation time is believed to give a representative measure of the biofilm forming abilities of the strains within the temperature range used during production [14, 34]. After incubation, the wells were washed with tap water, stained with 1% crystal violet solution (Sigma-Aldrich, St. Louis, MO, USA) for 30 min at room temperature, and washed at least three times with tap water to remove excess dye. The remaining dye was dissolved in ethanol-acetone (70:30, vol/vol) for 10 min at room temperature, and the absorbance at 595 nm (A_595_) (Multiscan MS; Thermo Fisher Scientific, Inc., Waltham, MA, USA) was measured. The assay was performed four times for all strains. In each assay, the results were calculated by subtracting the median A_595_ of the three parallel control wells (test broth only) from the median A_595_ of the three parallel sample wells. Finally, the mean A_505_ value of all four assays were calculated for each strain. A_595_ values higher than three standard deviations of the negative controls were classified as positive for biofilm production [35]. The results of different groups were compared using a Mann Whitney test if no other test is specified, and *p*-values ≤0.05 were considered statistically significant.

### Biofilm morphotyping

Biofilm morphotyping was performed as previously described methods with slight modifications [36]. In brief; 1 μL of overnight culture was inoculated onto CR agar plates, i.e. LB agar without NaCl containing 40 μg/mL Congo Red (Merck) and 20 μg/mL Coomassie brilliant blue (Sigma-Aldrich, St. Louis, MO). After inoculation, the CR agar plates were incubated at 20.0 ± 1.0 °C. All plates were visually examined after 2, 6 and 8 days of incubation, and the morphotypes were categorized as: RDAR - indicating expression of curli fimbriae and cellulose, PDAR - indicating expression of cellulose but not fimbriae, BDAR - indicating expression of fimbriae but not cellulose, and SAW - indicating expression of neither cellulose nor fimbriae.

### Mixed biofilm production on glass slides

Six strains, two of each of the morphotypes RDAR, BDAR and PDAR, were used for these experiments (Table 4). Twelve different pairs of the combinations RDAR + BDAR, RDAR + PDAR and BDAR + PDAR were tested (Table S1). For each pair, overnight cultures of the two strains were mixed in the ratios 30:70, 50:50 and 70:30. From each mixture, as well as from the overnight cultures of the single strains, 200 μL were inoculated into sterile centrifuge tubes (Sarstedt AG & Co KG, Nürnbrecht, Germany) containing 5 mL LB^wo^/NaCl. An autoclaved microscope slide (76 by 26 mm; (Menzel GmbH + CoKG, Braunschweig, Germany) was placed in each tube. The tubes were incubated at 20.0 ± 1.0 °C for 2 days. During incubation, biofilm was formed on both sides of the microscope slides at the liquid-air interface. After incubation, total cfu was enumerated in the planktonic phase in the growth medium by serial dilutions and spreading on CR agar plates. To enumerate the bacteria in the biofilms, these were washed three times in sterile saline to remove loosely adhered bacteria. Thereafter, the biofilms were removed by scraping with a sterile cell scraper (BD Falcon, Bedford, MA, USA) and transferred to sterile reagent tubes containing 5 mL sterile saline and 20 glass beads (3 mm; Assistent, Glaswarenfabrik Karl Hecht GmbH & Co KG, Bavaria, Germany). The tubes were vortexed at 2000 rpm for 1 minute before the solutions were serial diluted in sterile saline and spread on CR agar plates. All plates were incubated at 37.0 ± 1.0 °C for 24 h. After incubation, the number of cfu of each strain was counted. The strains were differentiated by their morphotype. All experiments were performed twice. A two-tailed Students’ t test were used in statistical analyses on these results, and *p*-values ≤0.05 were considered statistically significant.

### Mixed biofilm production on CR agar plates

The same strains, pairs and ratios as in the glass slide assay were used in the CR agar plate assay. When inoculating single strains, 1 μL of overnight culture was used. For each pair of strains, 1 μL from each mixture of overnight cultures was inoculated. All inoculations were made in parallel. The CR agar plates were incubated at 20.0 ± 1.0 °C for 10 days, and the plates were visually examined every day the first 4 days, and every third day thereafter. All experiments were performed twice.

## Supplementary information

**Additional file 1: Table S1.** The percentage and mean A_595_ of biofilm positive strains (A_595_ > 0.085) in the microtiter plate assay, in total material and within each morphotype. Means with different letters are statistically different (*p* ≤ 0.05). **Table S2.** Comparison of biofilm production by isolates from chicken caecal and retail meat samples, as indicated by A_595_ in the microtiter plate assay, in the total material and within phylotypes. **Table S3.** Pairs of strains (as indicated by X) used in studies on mixed biofilms in the glass slide assay and CR plate assay. **Table S4.** Mean total log_10_ cfu ± standard deviation in biofilm and planktonic phase the glass slide assay after incubation of single strains and pairs of strains. **Table S5.** Comparison of mean log_10_ cfu by each morphotype in single strain and mixed biofilms. The means are based on data from all combinations of strains and inoculation ratios. “BDAR” includes both BDAR strains, “PDAR” includes both PDAR strains, AND “RDAR” includes both RDAR strains. **Figure S1.** Distribution of morphotypes within each phylotype and in the total material.

## Data Availability

The datasets used and/or analysed during the current study are available from the corresponding author on reasonable request.

## Abbreviations

A: Absorbance
BDAR: Brown, dry and rough (biofilm morphotype)
CR: Congo Red
LB: Luria-Bertani broth
PDAR: Pink, dry and rough (biofilm morphotype)
QREC: Quinolone resistant *Escherichia coli*
RDAR: Red, dry and rough (biofilm morphotype)
SAW: Smooth and with (non-biofilm morphotype)
