# Supplementary material for: Biofilm forming properties of quinolone resistant Escherichia coli from the broiler production chain and their dynamics in mixed biofilms
Source: BMC Microbiol. 2020 Mar 4;20:46. doi: 10.1186/s12866-020-01730-w (PMC7055085; doi:10.1186/s12866-020-01730-w)

Supplementary material

Table S1. The percentage and mean A_{595} of biofilm positive strains ($A_{595} > 0.085$) in the microtiter plate assay, in total material and within each morphotype. Means with different letters are statistically different ($p \leq 0.05$).

Morpho-type	% biofilm positive	Biofilm positive mean A_{595} ± SD
RDAR	95.5	1.576^{A} ± 0.930
BDAR	59.5	1.011^{C} ± 0.765
PDAR	60.0	0.226^{B} ± 0.054
ALL	84.2	1.439 ± 0.933

Table S2. Comparison of biofilm production by isolates from chicken caecal and retail meat samples, as indicated by A_{595} in the microtiter plate assay, in the total material and within phylotypes.

	Chicken caecal samples			Chicken retail meat samples			
Phylo-type	No	%	Mean A_{595} ± SD	No	%	Mean A_{595} ± SD	p *
A	12	14.1	1.394 ± 1.135	9	12.3	1.279 ± 1.151	0.82
B1	12	14.1	1.050 ± 0.978	13	17.8	1.256 ± 0.670	0.54
B2	38	44.7	1.110 ± 0.929	36	49.3	1.148 ± 0.798	0.85
D	23	27.1	1.738 ± 1.179	15	20.5	0.756 ± 1.216	0.02
ALL	85	100.0	1.312 ± 1.057	73	100.0	1.103 ± 0.924	0.19

* Student's t-test, caecal samples vs retail meat samples

Table S3. Pairs of strains (as indicated by X) used in studies on mixed biofilms in the glass slide assay and CR plate assay.

	PDAR-1	PDAR-2	BDAR-1	BDAR-2
RDAR-1	X	X	X	X
RDAR-2	X	X	X	X
PDAR-1			X	X
PDAR-2			X	X

Table S4. Mean total $\log_{10}$ cfu ± standard deviation in biofilm and planktonic phase the glass slide assay after incubation of single strains and pairs of strains.

	Single strains	Pairs	Students t-test
In biofilm	5.63 ± 0.73	5.80 ± 0.30	$p = 0.17$
In planktonic phase	9.50 ± 0.18	9.28 ± 0.34	$p = 0.01$

Table S5. Comparison of mean $\log_{10}$ cfu by each morphotype in single strain and mixed biofilms. The means are based on data from all combinations of strains and inoculation ratios. "BDAR" includes both BDAR strains, "PDAR" includes both PDAR strains, AND "RDAR" includes both RDAR strains.

	Biofilm mean $\log_{10}$ cfu ± SD				
	BDAR	PDAR	RDAR-1	RDAR-2	RDAR
Single strain biofilms	4.75 ± 0.44	6.21 ± 0.14	5.82 ± 0.04	6.07 ± 0.16	5.94 ± 0.18
Mixed biofilms					
BDAR + PDAR	4.41 ± 0.26	5.71 ± 0.24 *			
BDAR + RDAR-1	4.24 ± 0.14***		5.52 ± 0.16**		
BDAR + RDAR-2	4.74 ± 0.22			6.06 ± 0.32	
BDAR + RDAR	4.49 ± 0.36				5.79 ± 0.35
PDAR + RDAR-1		5.31 ± 0.28 *	5.01 ± 0.40 *		
PDAR + RDAR-2		5.08 ± 0.29 *		6.07 ± 0.14	
PDAR + RDAR		5.21 ± 0.31 *			5.54 ± 0.62

* $p \le 0.01$, ** $p \le 0.05$, *** $p = 0.07$ (Student's t-test)

Figure S1. Distribution of morphotypes within each phylotype and in the total material

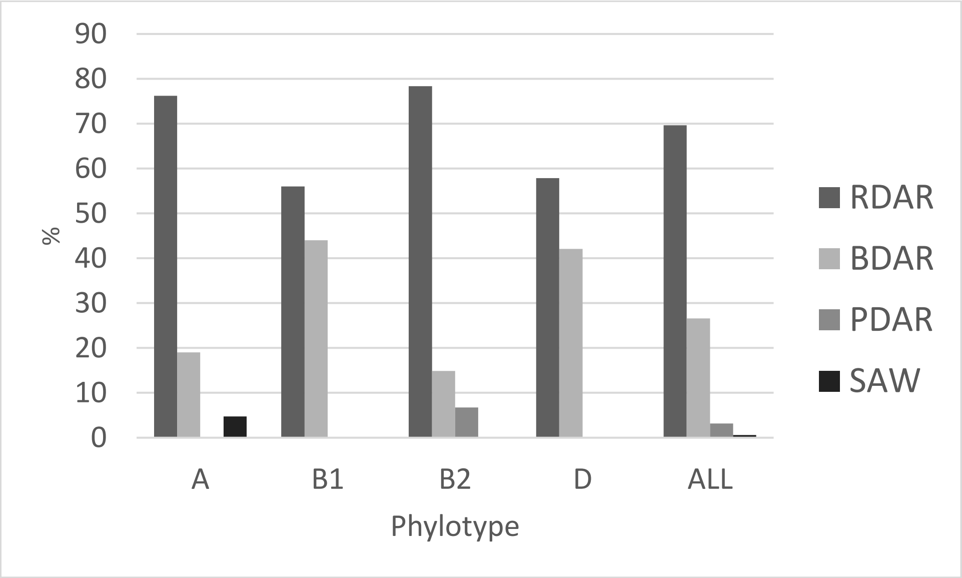

Supplement: Supplementary file 1 — Additional file 1: Table S1. The percentage and mean A595 of biofilm positive strains (A595 > 0.085) in the microtiter plate assay, in total material and within each morphotype. Means with different letters are statistically different (p ≤ 0.05). Table S2. Comparison of biofilm production by isolates from chicken caecal and retail meat samples, as indicated by A595 in the microtiter plate assay, in the total material and within phylotypes. Table S3. Pairs of strains (as indicated by X) used in studies on mixed biofilms in the glass slide assay and CR plate assay. Table S4. Mean total log10 cfu ± standard deviation in biofilm and planktonic phase the glass slide assay after incubation of single strains and pairs of strains. Table S5. Comparison of mean log10 cfu by each morphotype in single strain and mixed biofilms. The means are based on data from all combinations of strains and inoculation ratios. “BDAR” includes both BDAR strains, “PDAR” includes both PDAR strains, AND “RDAR” includes both RDAR strains. Figure S1. Distribution of morphotypes within each phylotype and in the total material. [file 12866_2020_1730_MOESM1_ESM.pdf]
